# Development of a 3D Brain Model to Study Sex-Specific Neuroinflammation After Hemorrhagic Stroke

**DOI:** 10.1007/s12975-024-01243-y

**Published:** 2024-04-01

**Authors:** Rezwanul Islam, Hadi Hasan Choudhary, Hritik Mehta, Feng Zhang, Tudor G. Jovin, Khalid A. Hanafy

**Affiliations:** 1 Department of Biomedical Sciences, Cooper Medical School at Rowan University, Camden, NJ USA; 2 Cooper Neurological Institute, Cooper University Health Care, Camden, NJ USA; 3 Center for Neuroinflammation, Cooper Medical School at Rowan University, Camden, NJ USA

**Keywords:** 3D cell culture, Cerebrospinal fluid, Subarachnoid hemorrhage, Neuroinflammation, Microglia, Astrocytes, Neurons, Sex-specific

## Abstract

Subarachnoid hemorrhage (SAH) accounts for 5% of stroke, with women having a decreased inflammatory response compared to men; however, this mechanism has yet to be identified. One hurdle in SAH research is the lack of human brain models. Studies in murine models are helpful, but human models should be used in conjunction for improved translatability. These observations lead us to develop a 3D system to study the sex-specific microglial and neuroglial function in a novel in vitro human SAH model and compare it to our validated in vivo SAH model. Our lab has developed a 3D, membrane-based in vitro cell culture system with human astrocytes, microglia, and neurons from both sexes. The 3D cultures were incubated with male and female cerebrospinal fluid from SAH patients in the Neuro-ICU. Furthermore, microglial morphology, erythrophagocytosis, microglial inflammatory cytokine production, and neuronal apoptosis were studied and compared with our murine SAH models. The human 3D system demonstrated intercellular interactions and proportions of the three cell types similar to the adult human brain. In vitro and in vivo models of SAH showed concordance in male microglia being more inflammatory than females via morphology and flow cytometry. On the contrary, both in vitro and in vivo models revealed that female microglia were more phagocytic and less prone to damaging neurons than males. One possible explanation for the increased phagocytic ability of female microglia was the increased expression of CD206 and MerTK. Our in vitro, human, 3D cell culture SAH model showed similar results to our in vivo murine SAH model with respect to microglial morphology, inflammation, and phagocytosis when comparing the sexes. A human 3D brain model of SAH may be a useful adjunct to murine models to improve translation to SAH patients.

**Supplementary Information:**

The online version contains supplementary material available at 10.1007/s12975-024-01243-y.

## Introduction

While SAH accounts for only 5% of all stroke cases, the morbidity is high due to the relatively young age of those afflicted [1, 2]. No treatment to date addresses the persistent red blood cell (RBC) burden and concomitant inflammation induced by the extravasated RBCs. In mouse models of hemorrhagic stroke, our lab and others have shown that microglia (MG) derived from adult mice are necessary for the initiation and propagation of RBC-induced cerebral inflammation [3–7]. One of the critical MG receptors responsible for initiating RBC-induced cerebral inflammation is the toll-like receptor 4 (TLR4). TLRs are preferentially expressed on antigen-presenting cells like macrophages, and heme in RBCs specifically activates macrophage TLR4 [5, 8–10]. Classically, TLRs respond to infectious pathogen-associated molecular patterns (PAMPs); no such infectious agent exists in hemorrhagic stroke [5, 8–11]. In mouse models of hemorrhagic stroke, MG TLR4 responds to the breakdown products of RBCs to initiate cerebral inflammation.

Furthermore, whether mouse or human, almost no research has been done on sex-specific signal transduction of subarachnoid hemorrhage [12, 13]. Even though women with SAH are older, have more aneurysms, and are twice as likely as men to have an SAH, outcomes are similar or superior in women compared to men [14–17]. This sex difference in outcome is unexpected because mixed cohorts, where males and females were analyzed as a group, suggest that increasing age and aneurysm number are independent risk factors for a poor outcome [18–20]. Therefore, females with SAH might have protective factors that, despite an increased disease burden, can still maintain outcomes similar, or superior, to males. A recent meta-analysis in stroke and the new AHA guidelines on SAH support this finding [21, 22]. Furthermore, a recent study in JAMA confirms that female participants continue to be underrepresented in many disease categories, including neurology (stroke); thus, current data about outcomes in this field need further study and more power to detect differences between the sexes [23].

We set out to investigate sex differences in SAH using both a novel 3D human brain in vitro model and our murine SAH model. Classically, in vitro microglia culture models ignore the effects of other neuroglia, and the microglia in 2D culture have very different characteristics from in vivo models [24]. For this reason, we set out to develop a more accurate 3D human culture system that accounts for the effects of some of the other neuroglia, and the intercellular interactions that occur in a natural 3D environment as opposed to 2D.

Furthermore, to study sex-specific outcomes in SAH, we compared our 3D in vitro human model using male and female human microglial, astrocytic, and neuronal cell lines, incubated with male and female cerebrospinal fluid from SAH patients in the Neuro-ICU, respectively. Moreover, these results were compared with male and female murine SAH models in microglial morphology, erythrophagocytosis, microglial inflammatory cytokine prediction, and neuronal apoptosis. In both the in vivo murine models and our novel 3D human brain in vitro model, female microglia and the female cerebral milieu were less inflammatory, more phagocytic, and less prone to neuronal damage than their male counterparts.

## Materials and Methods

### ARRIVE and Good Research Practice

The outlined study conforms to ARRIVE guidelines to ensure transparency, reproducibility, and ethical conduct. Throughout this manuscript, we have provided comprehensive descriptions of our experimental design, methods employed, animal characteristics, housing conditions, and welfare considerations. Specifics on sample sizes, randomization, blinding techniques, and statistical analyses are reported. Researchers performing the murine SAH procedure were not involved in the subsequent analysis of these mice, thereby ensuring unbiased results. Similarly, researchers initially involved in plating the male and female 3D human brain cultures were not involved in the analysis.

### Animals

Eight- to ten-weeks-old adult male and female C57BL/6 (Stock 000664) and CD45.1 BL/6 (Stock 002014) mice were purchased from Jackson laboratory and were housed under pathogen-free conditions, exposed to a standard 12-h day-night light cycle, and received standard chow ad libitum. All animal experiments were approved by the Cooper University IACUC (23–015).

### Cells

To generate a 3D culture, human astrocyte, neuron, and microglia cells were grown on an Alvetex scaffold (Reprocell, Cat# AVP004) [25]. The human male microglial cells were purchased from Accegene (ABC-TC3704), and female microglial cells were bought from BrainXell (BX-0900e-32). Male neuron progenitor cells (ACS 5004) and female neuron (CRL-3592) were purchased from ATCC. Male astrocytes were purchased from Cellular Dynamics (C1249), and female astrocytes were purchased from ATCC (CRL-1718). Before being grown together on the Alvetex membrane, all cells were grown in individual cell cultures for 1 week. Male neurons were grown in DMEM: F12 (ATCC 30–2006) supplemented with the Growth Kit for Neural Progenitor Cell Expansion (ATCC ACS-3003) [26]. Female neurons were grown in high glucose DMEM (VWR, Cat# 76,470–182) supplemented with 4 mM L-glutamine adjusted to contain 1.5 g/L sodium bicarbonate, 4.5 g/L glucose, 10% fetal bovine serum, (Avantor, Cat# 76,419–584), and 1X antibiotic antimycotic mixture (HyClone, Cat# SV30079.01) [27]. Male microglia were grown in manufacturer-provided medium, ABM-TM3704 Human Microglia Medium [28]. Female microglia were grown in a microglia basal medium with culture supplements [29]. Male astrocytes were grown in iCell human astrocyte 2.0 medium [30]. Female astrocytes were grown in ATCC-formulated RPMI-1640 Medium, (ATCC 30–2001) supplemented with fetal bovine serum (ATCC 30–2020) to a final concentration of 10% and 1X antibiotic antimycotic mixture [31]. When grown together on the Alvetex scaffold in 3D cell culture, all cells were maintained in high glucose DMEM supplemented with 10% fetal bovine serum and 1X antibiotic antimycotic solution.

### Study Population

This study is an investigator-initiated, prospective, observational, cohort study of aneurysmal SAH patients admitted to the neurological intensive care unit (neuro-ICU) at the Cooper University Hospital in Camden, New Jersey. With informed consent from the patients or their legally authorized representative (LAR) approved by the Cooper University Institutional Review Board (23–099), CSF was collected from patients who had external ventriculostomy drains (EVDs) placed for clinical reasons within 48 h of ictus. The diagnosis of SAH was established by computed tomography (CT) and CT angio and then confirmed by a 4-vessel angiogram when the aneurysm was secured. All CSF collected in this study was from SAH patients who were coiled. Patients were not enrolled if (1) they were less than 18 years old; (2) they were pregnant; (3) more than 48 h had elapsed since ictus; or (4) they, their families, or LARs did not provide consent for participation in the study. All patients who qualified for the study and provided consent for participation or had such consent provided by their LARs were included. All patient eligibility requirements, consent methodology, and enrollment protocols, as well as sample collection, processing, and storage procedures were approved by the Cooper IRB.

### CSF Collection and Cell Pellet Harvest

Twenty milliliters of CSF was collected from each SAH patient in a sterile manner from the most distal EVD port within 48 h of ictus. The collected CSF was transported on ice and immediately processed. Sixty microliters of CSF was used in the 3D human cell culture, and the remaining CSF was centrifuged at 500 g for 5 min; the resulting red blood cell (RBC) pellet from the CSF was then used for the in vitro phagocytosis assay explained in the “Flow Cytometry” section below.

### 3D Brain Culture on Alvetex Scaffold with Cerebrospinal Fluid from SAH Patient

Alvetex scaffolds were placed in inserts of 6 well plates for our study. The scaffold was activated by incubating with 75% ethanol for 10 min and then coated with 1.5 µg/ml Poly-L-ornithine solution for 24 h. Following activation, astrocytes were seeded onto the scaffold (2.5 × 10^6^ cells/well) and grown for 7 days, prior to the addition of neurons (80 k cells/well) and microglia (5 k cells/well), each added at opposing edges of the scaffold. Cells were allowed to grow together for 3–4 days. Finally, 60 µl of CSF from one SAH patient was added to one 3D brain culture. For “sham” 3D brain culture, 60 µl of PBS was added. The experiments were repeated in triplicate, and the sex of the CSF sample was matched to the sex of the 3D brain culture.

### In Vivo* Murine SAH*

Surgery to induce SAH in our mouse model has been tested and validated [32–34]. Briefly, mice were anesthetized by intraperitoneal administration of a Ketamine (110 mg/kg)-Xylazine (5 mg/kg) cocktail. Following appropriate anesthesia, the cranium was fixed in a stereotaxic frame (Kopf Instruments, Tujunga, CA). A midline incision was performed to visualize the skull. A burr hole was then drilled along the midline, 4.5 mm anterior to the bregma. With a burr hole in place, 60 µl of arterial blood from a CD45.1 BL/6 donor mouse was collected by cardiac puncture. This arterial blood was then injected over a 10-s period, through the burr hole using a 27-gauge needle (BD Needles, Cat# 405,081), at a 40° ventral angle at the level of the skull base, which was 4.5 to 5 mm dorsoventral. The spinal needle was left in place for 3 min to prevent backflow of blood. Bone wax was then used to close the burr hole, and a 5–0 mattress suture was used to approximate the epidermis and close the incision.

### Flow Cytometry

*In 3D cell culture:* For analyzing 3D cell culture by flow cytometry, cells were retrieved from the scaffold by trypsin digestion and then incubated with human TruStain FcX Fc-receptor blocker (1:50, BioLegend, Cat# 422,302) to block non-specific sites. Cells were incubated with BV421-CX3CR1 (1:100, BioLegend, Cat#341,620), AF-700-CD45 (1:100, BioLegend, Cat# 304,024), FITC-Tmem119 (1:100, Abcam, Cat# ab225497), APC-β-III-tubulin (1:100, BD Biosciences, Cat# 558,606), PE-GFAP (1:100, BD Biosciences, Cat# 561,483), and APC-CY7-CD11b (1:100, BioLegend, Cat# 101,226) fluorophore‐conjugated anti-human monoclonal antibodies. Astrocytes and neurons were identified by GFAP^hi^ and β-III-tubulin^hi^ populations, respectively, gated on all cells. Microglia were defined by sequential gates with a CD11b^hi^CD45^med^ signature, followed by a Tmem119^hi^Cx3Cr1^med^ signature, from the non-astrocyte, non-neuron population (Supplementary Fig. 1).

For the in vitro microglial phagocytosis assay, 3D cell culture was incubated with pHrodo™ Red (Invitrogen, Cat# P35372) only (sham) or RBC-tagged-pHrodo (SAH). For RBC-pHrodo labeling, the RBC pellet from human CSF was washed with PBS and treated with pHrodo for 30 min in the dark, due to its light-sensitive nature. RBC-pHrodo was then added to the 3D cell culture and incubated for 3 h at 37 °C, 5% CO2, followed by flow cytometric analysis. The microglial population was gated as above, and phagocytosis was assessed by measuring microglia that were pHrodo^+^.

For the intracellular cytokine assay, 3D cell culture was exposed to a cell stimulation cocktail (Invitrogen 00497093) for 5 h. Cells were then stained with microglial markers, as above, (30 min), fixed with 4% PFA (15 min), permeabilized with 0.1% tween in PBS (15 min), washed with PBS, and stained with intracellular cytokine marker, PE-CY7-IFNγ (1:100, BD Biosciences, Cat# 557,643). Flow analysis as above. Gating strategy is shown in supplementary Fig. 2A.

*In mouse brain:* CNS immune cells from the mouse were isolated using the debris removal solution (Miltenyi Biotec, Cat# 130–109-398). Cells were washed and incubated with mouse Fc-receptor blocker (1:50, BioLegend, Cat# 101,320) to prevent non-specific binding and stained with APC-CY7-CD11b (1:100, BioLegend, Cat# 101,226), PB-CD45 (1:100, BioLegend, Cat# 103,126), AF700-CD45.1 (1:100, BioLegend, Cat# 110,724), APC-CX3CR1 (1:100, BioLegend, Cat# 149,008), and PE-Tmem119 (1:100, Invitrogen, Cat# 12–6119-82) using fluorophore‐conjugated monoclonal antibodies. Compensation experiments were conducted using beads before sample data acquisition, following previously published protocols [35]. To distinguish between endogenous leukocytes and those injected from the donor mouse, CD45.1^+^ cells were excluded and subsequent analysis of only CD45^+^ cells was performed. Microglia were identified by CD11b^hi^CD45^med^Tmem119^hi^Cx3Cr1^med^ population gated off the parent monocyte population. For the in vivo phagocytosis assay, the cells were fixed, permeabilized, and stained intracellularly with FITC-Ter-119 (murine RBC) (1:100, BioLegend, Cat# 116,206). An inflammatory cytokine production assay was done with PE-CY7-IFNγ (1:100, Tonbo Biosciences, Cat# TB-60–7311-U025) intracellular staining of fixed CNS cells, and microglia were characterized as mentioned above. Microglial phagocytosis and cytokine production were measured by Ter119^+^ and IFNγ^+^ microglial populations, respectively. Data was analyzed on FlowJo. Gating strategy is shown in supplementary Fig. 2B.

### Immunohistochemistry

*In mouse brain:* Immunostaining assay was performed as described previously [36]. Mouse brains were fixed with 4% PFA by cardiac perfusion and then collected and embedded in OCT. Fresh frozen brains were cryosectioned into 10 µm serial coronal sections with a Leica CM3050 S cryostat. Next, the brain slices were permeabilized with 0.1% Tween-20 in PBS, blocked with 10% normal goat serum for 1 h at RT, and stained overnight at 4 °C with primary antibodies. We performed double staining with rabbit monoclonal β-III-tubulin (1:500, Cell Signaling Tech. Cat# 5568) and mouse monoclonal TMEM119 (1:500, Cell Signaling Tech. Cat# 41,134) to identify neurons and microglia, respectively. Additionally, mouse monoclonal GFAP (1:500, Cell Signaling Tech. Cat# 3670) and rabbit monoclonal TMEM119 (Cell Signaling Tech. Cat# 90,840) antibodies were used to astrocytes and microglia, respectively. For detecting microglial phagocytosis of erythrocytes, cells were stained with anti-mouse Ter119 (murine erythrocytes) (1:300, BioLegend, Cat# 1,116,202) and rabbit monoclonal TMEM119 (microglia) (Cell Signaling Tech. Cat# 90,840) antibodies. Further, mouse brains were stained with anti-mouse MerTK (1:50, BioLegend, Cat# 151,523) and anti-mouse CD206 (1:50, BioLegend, Cat# 141,732). Anti-rabbit-488 (1:500, Thermo Fisher Scientific) and anti-mouse-555 (1:500, Thermo Fisher Scientific) secondary antibodies were subsequently added.

*In 3D cell culture:* For immunostaining of the 3D cell culture, the membranes were washed with PBS and fixed in 4% PFA for 20 min at RT. The fixed membranes were embedded in OCT overnight and cryosectioned into 20 µm slices. For visualization, cells were permeabilized with 0.1% Tween-20 in PBS and stained with rabbit monoclonal β-III-tubulin (1:500, Cell Signaling Tech. Cat# 5568), mouse monoclonal TMEM119 (1:500, Cell Signaling Tech. Cat# 41,134), and goat polyclonal GFAP (1:500, Novus Biologicals, Cat# NB100-53809) antibodies. For detecting phagocytosis of erythrocytes, cells were stained with CD235a (1:500, Cell Signaling Tech. Cat# 75,126) (human erythrocytes) and mouse monoclonal TMEM119 (1:500, Cell Signaling Tech. Cat# 41,134) antibodies, MerTK (1:50, BioLegend, Cat# 151,523), CD206 (1:50, BioLegend, Cat# 141,732), followed by secondary staining with anti-rabbit-488 (1:500, Thermo Fisher Scientific), anti-mouse-555 (1:500, Thermo Fisher Scientific), and anti-goat-680 (1:500, Thermo Fisher Scientific).

*TUNEL assay:* For in vitro and in vivo terminal deoxynucleotidyl transferase dUTP nick end labeling (TUNEL) staining, fixed brain slices were permeabilized with 0.1% Triton X-100 for 2 min. After washing with PBS, sections were stained (In Situ Cell Death Detection Kit, TMR red; Roche Life Science, Cat# 12,156,792,910) for 1 h at 37 °C in a humidified atmosphere and mounted with DAPI.

*Confocal imaging:* All imaging was done with a Nikon A1R HD25 inverted confocal microscope. Z-stacks were analyzed and quantified using ImageJ software. Co-localization histograms were generated using the coloc-2 feature of ImageJ software. In brief, the program combines the green microglial single channel-1 saturation pixels with the red RBC single channel-2 saturation pixels to calculate the level at which they overlap. The y-axis represents channel-1 pixel intensity, while the x-axis represents channel-2-pixel intensity. Blue represents the lowest population overlap frequency possible, while yellow represents the highest. The lower the slope for each heat map, the more RBC staining there is per microglial staining. Lab personnel interpreting the immunostaining were not aware of the groups from whence they came.

### Calculation of Microglial Morphology

Microglial morphology was quantified using the Microglial Morphology Analysis Index (MMAI). MMAI was calculated by dividing the cell body area by the microglial pseudopodia area, in ImageJ. First, multichannel z-stacks (in vitro 3D cell culture) and 2D images (in vivo mouse brain slices) were split into individual channels. Regions of Interest (ROIs) were drawn using a free-hand selection tool. Two ROIs were drawn for every microglia: one along the cell body and another enclosing the pseudopodia projections. Measuring parameters were set to measure area and standard deviation in the set measurement tool in the Analyze menu, and the area was measured using the measure tool. Cell body area was subtracted from projection areas to get the area of pseudopodia alone. MMAI is calculated as the ratio between cell body area to pseudopodia area.

### Statistical Analysis

Continuous variables were assessed for normality with skewness and kurtosis. Data not normally distributed were reported as medians with interquartile ranges (IQRs). Data that were normally distributed were reported with means and standard deviations. To study the independent associations, we first performed a univariate chi-square analysis for categorical variables and a Student *t*-test for continuous variables. Only variables with *p* < 0.05 were considered statistically significant. Sample size was determined based on our previous work using the mean and common standard deviation. Multiple experimental groups were compared with repeated measures of one-way analysis of variance (ANOVA) with Bonferroni’s post hoc tests. The results are presented as mean ± SD for all experiments. Surgeries were performed at the same time of the day to eliminate circadian variation and minimize confounders. All statistical analyses were performed with SPSS 29 (IBM Corp.).

## Results

### 3D Cell Culture Model Generation

**Fig. 1:**
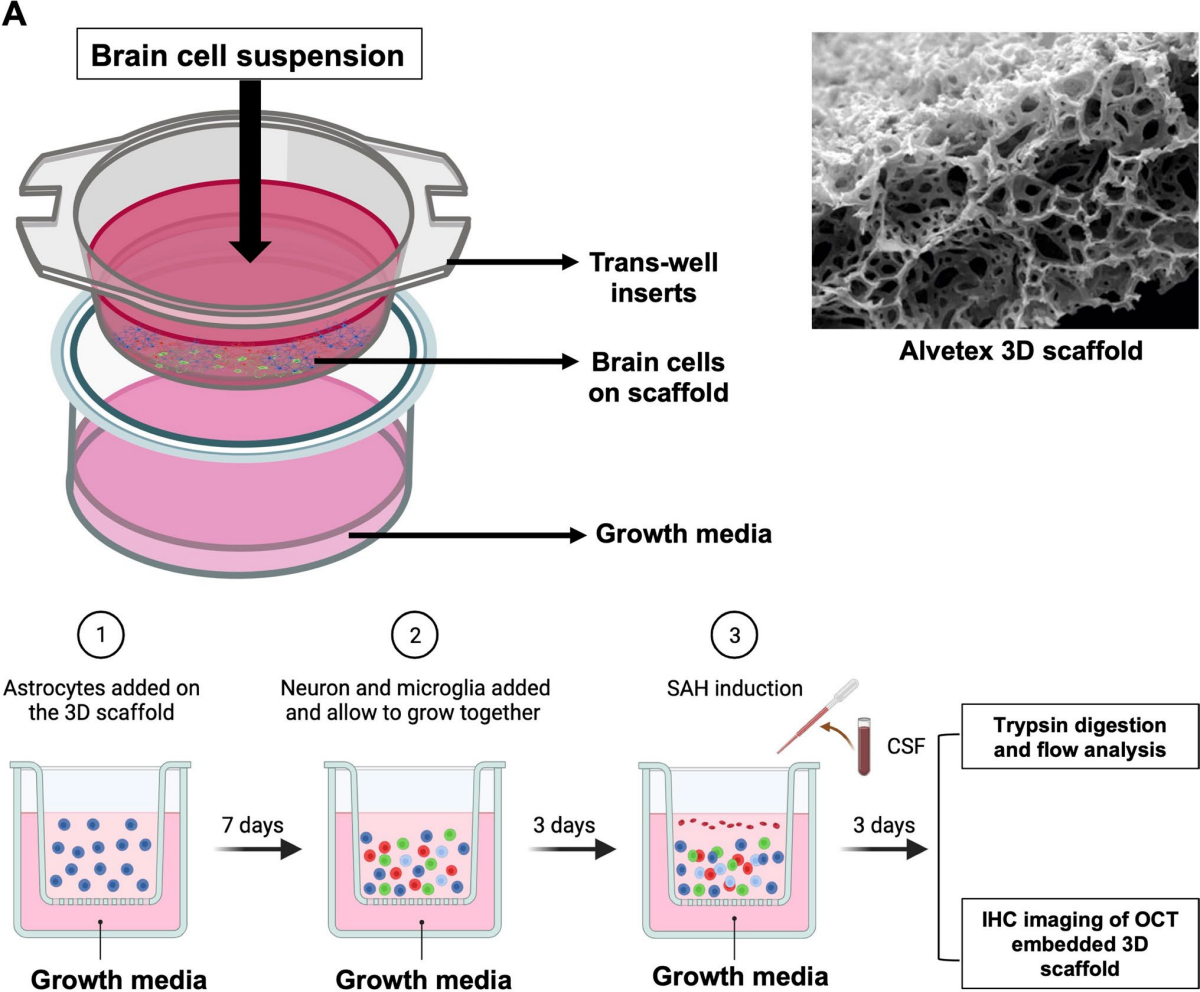

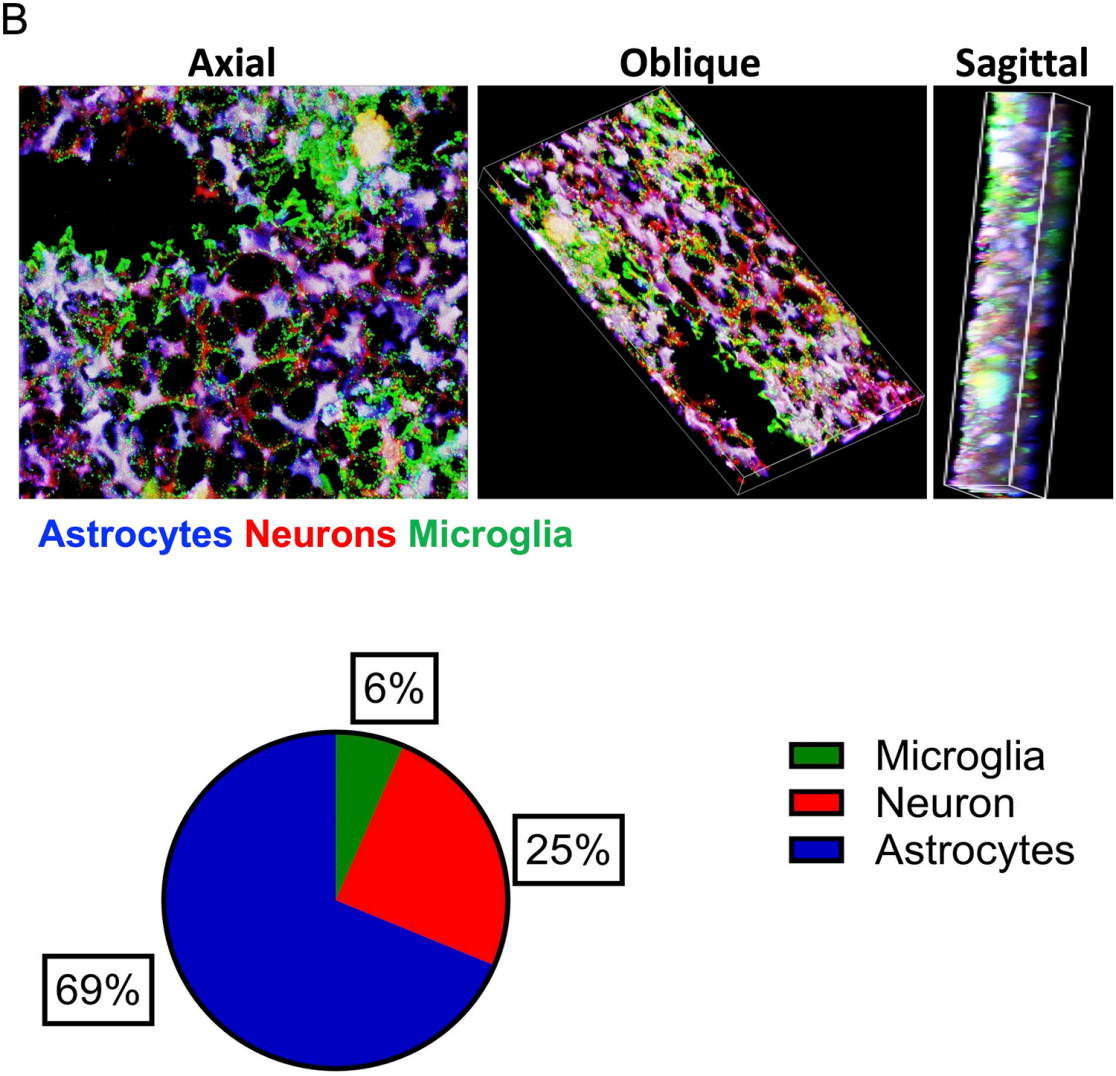
Construction of 3D model: **A** Schematic image representing the steps for constructing an in vitro brain unit using an Alvetex 3D scaffold. Figure created with BioRender.com. **B** Immunohistochemistry (IHC) of microglia, astrocytes, and neurons in 3D in vitro culture systems. Axial, oblique, and sagittal views are shown. Microglia, astrocytes, and neurons constitute 6%, 69%, and 25% of the brain, respectively. The pictures were taken on a Nikon Eclipse Ti confocal microscope and quantified on ImageJ software

Our 3D cell culture was seeded onto Alvetex scaffolds and grown on trans-well inserts to maintain adequate oxygenation and nutrient supply to all cells regardless of location (Fig. 1A). The timeline for how the experiments were conducted with respect to the addition of each of the 3 individual cell lines followed by the addition of sex-matched CSF is shown as well (Fig. 1A). The 3D cell culture retains the cellular ratios found in adult human brain: 69% astrocytes, 25% neurons, and 6% microglia (Fig. 1B).

### Patient Demographics for CSF Used to Generate 3D Brain SAH and Control Models

Table 1 describes the patient characteristics of the 4 male and 4 female SAH patients that were used for the 3D brain culture. Sham 3D brain cultures were treated with an equal volume of PBS, instead of CSF.

**Table 1:** Patient characteristics

Characteristics	SAH-male	SAH-female
No. of patients	4	4
Age in years	48 (42–55)	53 (49–62)
Hunt and Hess scale	4 (3–5)	3 (2–3)
mF grade	4 (3–4)	3 (2–3)
mRS score	3 (2–4)	2 (1–3)
History of smoking	3 (75%)	1 (25%)

### In Vitro* and *In Vivo* Brain Cell Interaction and Microglial Morphological Analysis*

**Fig. 2:**
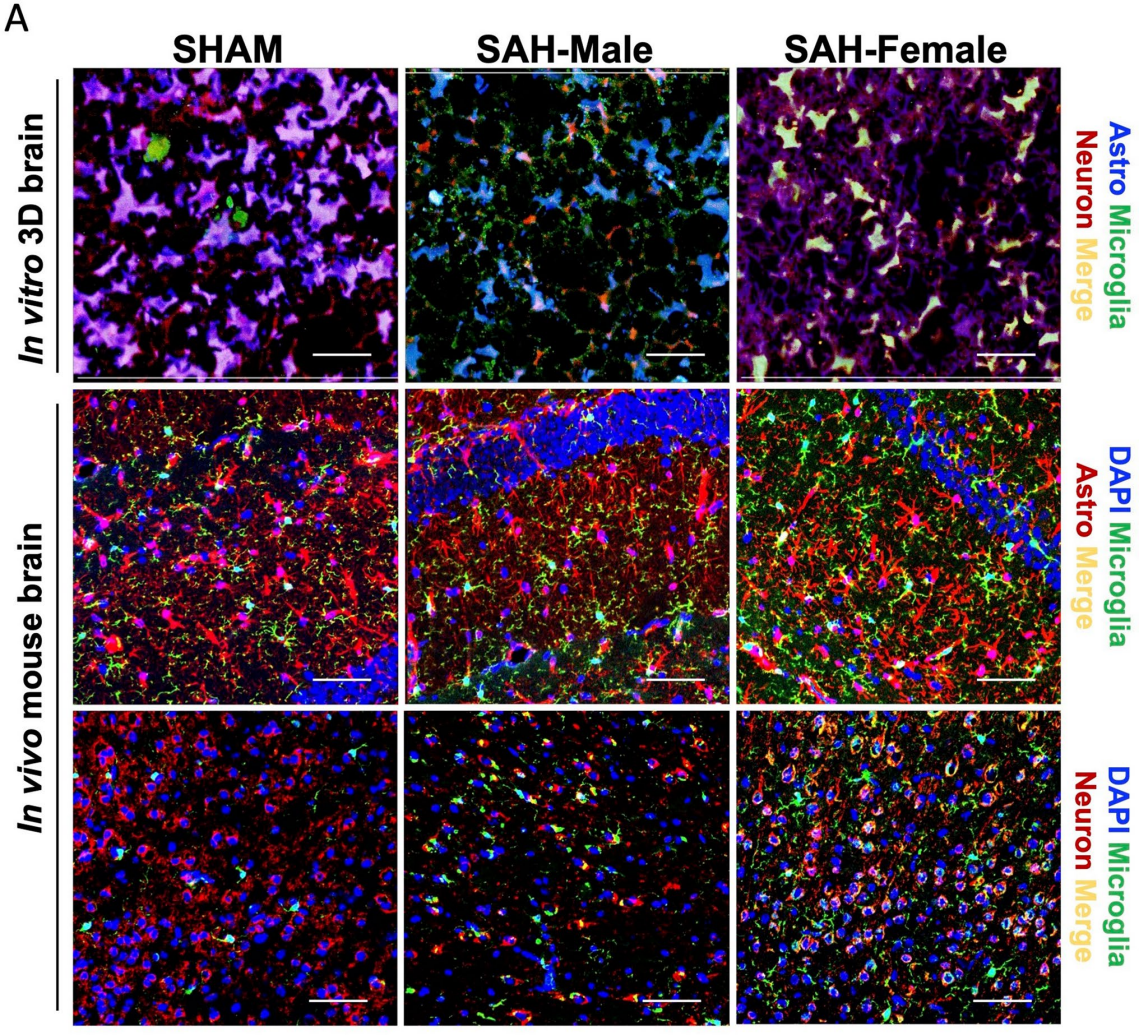

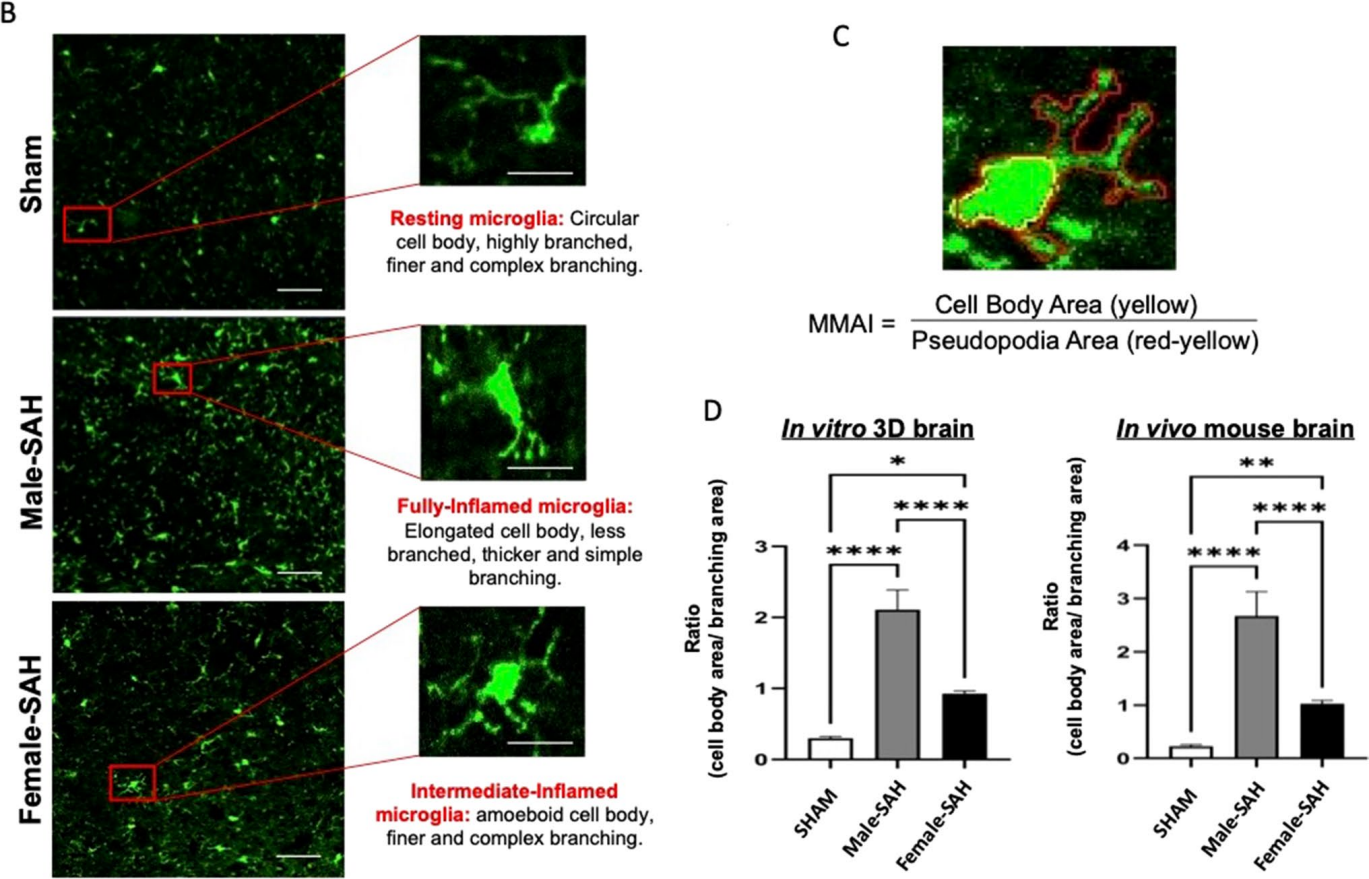
In vitro and in vivo SAH models: **A** IHC images showing the three major brain cells in sham and SAH (male and females) groups both in 3D and mouse brains. Immunostaining of the 3D brain (1st row): astrocytes (blue), neuron (red), and microglia (green); mouse brain (middle row): DAPI (blue), astrocytes (red), and microglia (green); mouse brain (bottom row): DAPI (blue), neuron (red), and microglia (green). Scale bar: 10 µm. **B** Quantification of MG-morphology: pseudopodia area (red) and cell body (yellow); the ratio of cell body area to branch area was calculated and termed microglial morphology analysis index (MMAI). Area measurements were done using ImageJ software. Statistical analysis was done by one-way ANOVA, *P < 0.05; *n* = 15 per group

Immunohistochemistry (IHC) images of the 3D brains showed the growth and interactions among the three cell types: astrocytes (blue), neuron (red), and microglia (green) in both conditions, sham and SAH (Fig. 2A-first row). No differences were seen between male and female 3D brain sham cultures, and so they were combined into one group throughout the manuscript as sham. IHC of the mouse brains also displayed similar cell–cell interactions: microglia (green) and astrocyte (red) (Fig. 2A-middle row); microglia (green) and neuron (red) (Fig. 2A-bottom row). Qualitatively, the morphology of the cells in the 3D culture sham and SAH was like those in the in vivo mouse model sham and SAH, respectively.

Quantitatively, microglia in 3D culture SAH and in vivo mouse model SAH revealed significantly bulkier cell bodies and stunted branching compared to 3D culture and in vivo shams, which showed a quiescent phenotype with small round cell bodies and long branches (Fig. 2B). We quantified these changes via the microglial inflammation analysis index (MMAI), which we defined as the cell body area divided by the pseudopodia area (Fig. 2C). We observed a threefold (in vivo) and sevenfold (in vitro) increase in MMAI in male SAH models compared to shams. In females, we observed a 2.5-fold (in vivo) and threefold (in vitro) increase in MMAI in female SAH models compared to shams. Male SAH exhibited a 2.6-fold (in vivo) and 2.3-fold (in vitro) increase in MMAI over female SAH. These data suggest that the MMAI correlates with inflammation because greater values are seen in SAH in both the 3D cell culture and the in vivo model when compared to their respective shams. Moreover, male SAH across both model systems consistently yield higher MMAI values than female SAH (Fig. 2D).

### 3D Brain Cells Characterization by Flow Cytometry

**Fig. 3:**
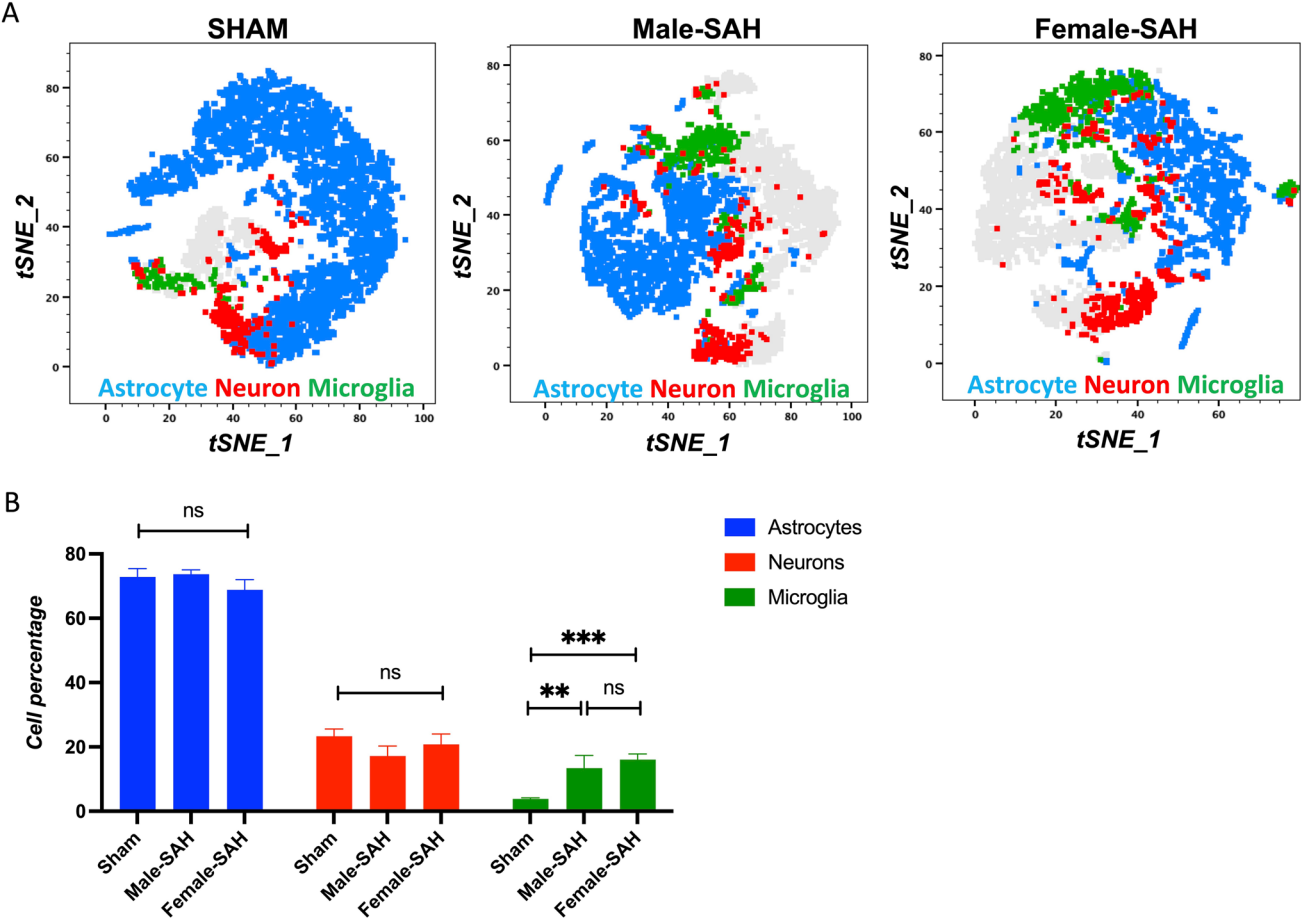
Flow cytometric analysis of 3D brain cells. **A** The tSNE plots created by FlowJo based on the flow results display the cell clusters: astrocyte (blue), neuron (red), and microglial (green) population of the 3D brain models. Scale bars = 20 µm. **B** The cell numbers found from the flow analysis were presented as the pie charts, created with GraphPad Prism, **p <0.01,***p <0.001

To support the notion that MMAI does in fact correlate with inflammation, we performed quantitative analysis by flow cytometry. First, we set out to determine if the number of glial cells in each 3D cell culture was different after incubation with CSF from SAH patients. Interestingly, the 3D cell culture revealed no significant difference in the neuroglial population, except in the microglial population where SAH was greater than sham. Of note, no difference was seen between the number of microglial in male and female 3D SAH culture (Fig. 3B). By tSNE plot, the activation of microglia in 3D cell culture, compared to sham, can be seen by the relative location of the population. Flow cytometry reveals that the microglia in SAH are characterized by a Tmem119^hi^CX3CR1^med^ signature, whereas in sham, the microglia are Tmem119^hi^CX3CR1^lo^ (Fig. 3A).

### Microglial IFNγ Production

**Fig. 4:**
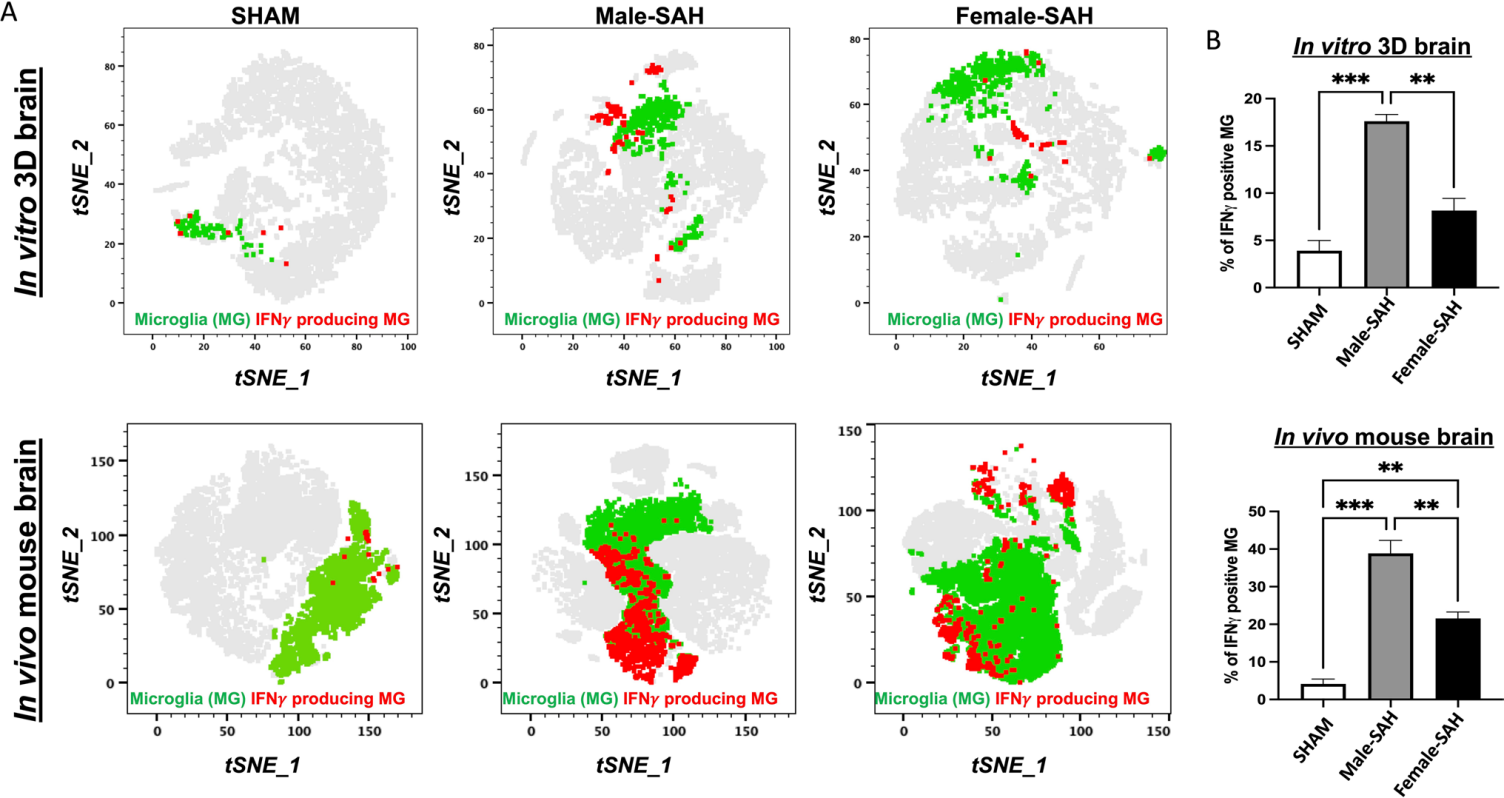
Flowcytometric analysis to measure inflammatory cytokine production by the microglia. **A** The representative tSNE plots indicate the microglia (green) and IFNγ producing MG population (red). Plots created by FlowJo. **B** Quantification of percent IFNγ producing MG. Statistical analysis revealed a significant increase in IFNγ production by the male MG than the females after SAH. Statistical analysis was done by one-way ANOVA, *P < 0.05; *n* = 4 per group, **p <0.01, ***p <0.001

A more definitive relationship between increased MMAI and inflammation can be established by measuring inflammatory cytokines specifically produced by microglia via flow cytometry. We chose to measure IFNγ because it is essential for the microglial phagocytosis of red blood cells and the critical response element of the heme-TLR4 pathway [37]. The representative tSNE plots reveal increased IFNγ production by microglia, more so in SAH, across both models compared to sham (Fig. 4A). Furthermore, quantified increased IFNγ production is greater in male SAH, across both models compared to females (Fig. 4B); thus, supporting the sex-specific MMAI findings.

### Red Blood Cell (RBC) Phagocytosis by Microglia

**Fig. 5:**
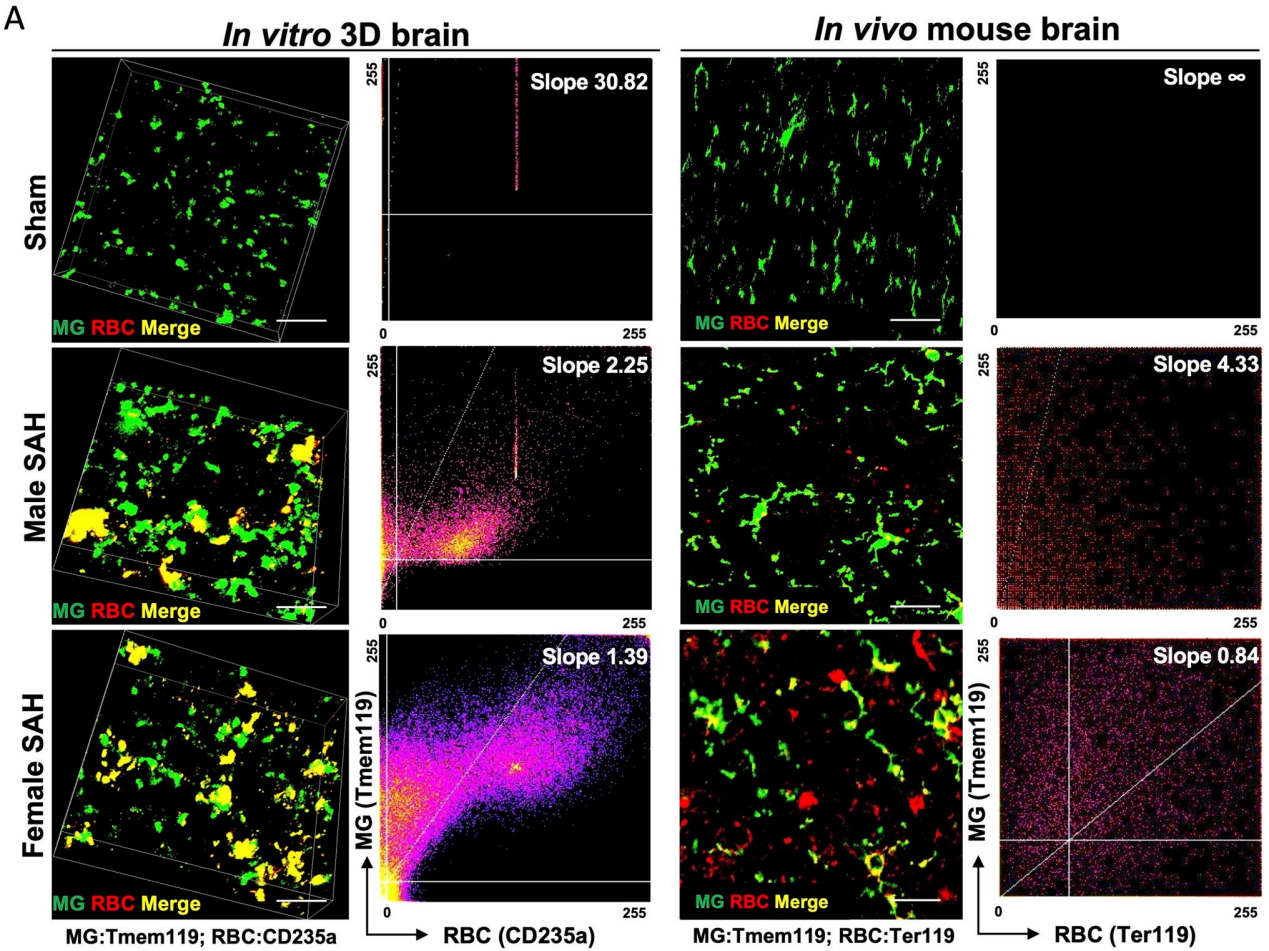

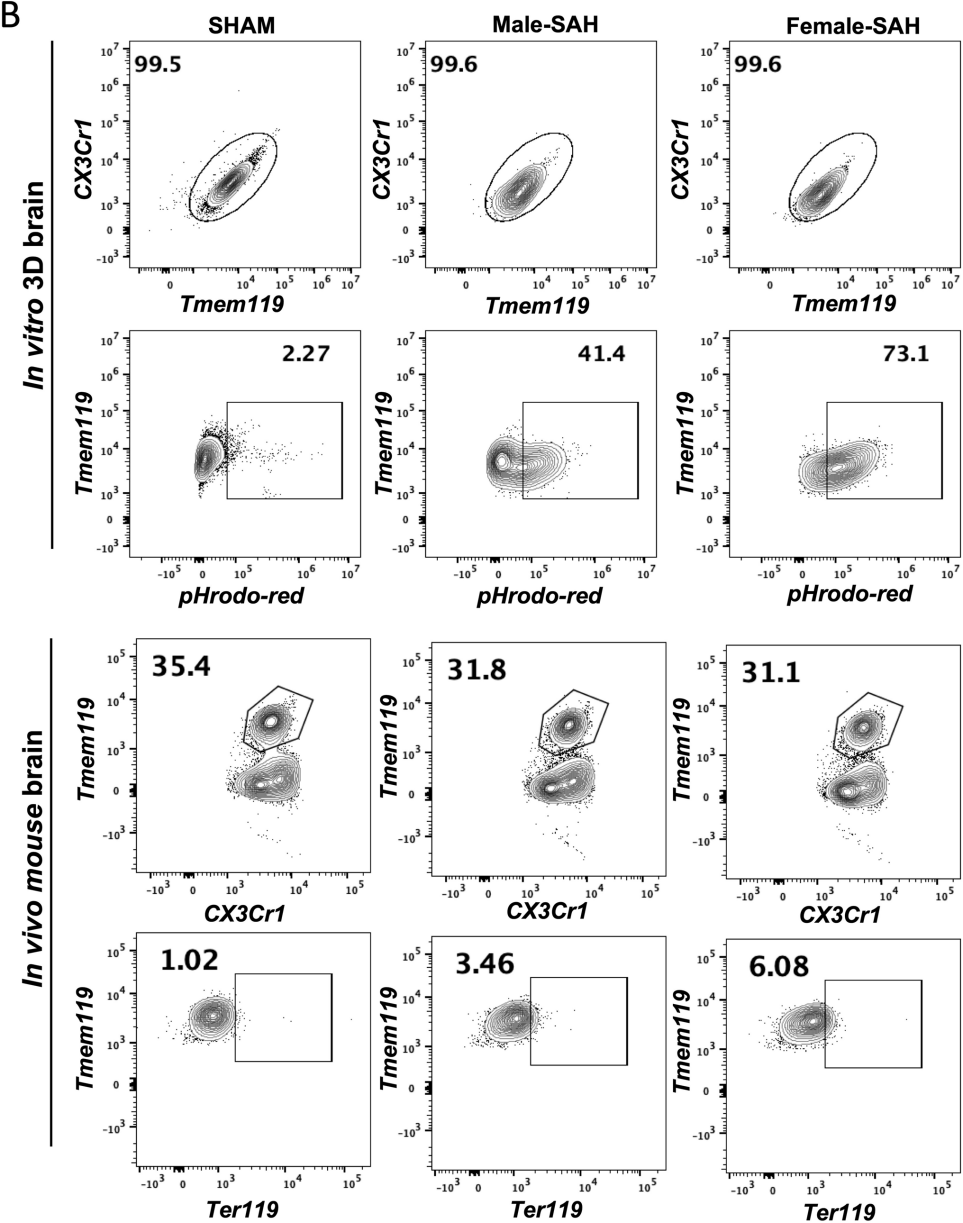
In vitro and in vivo phagocytosis assay*.* **A** The 3D brain cells stained with CD235a (red) + TMEM119 (green) and mouse brains with Ter119(red) + Tmem119 (green). Colocalization slope for each group was quantified using ImageJ software. The *x* and *y* axes represent pixel intensities for RBC (235a/Ter119) and MG (TMEM119), respectively. Lower slope means more RBC staining per microglia staining, and yellow color represents the highest possible frequency. Scale bars = 20 µm. **B** Flowcytometry-based phagocytosis assay with the in vitro and in vivo brain cells. In in vitro 3D SAH model, RBCs were labeled with pHrodo-Red (Invitrogen) and phagocytosis measured by Tmem119^+^pHrodo^+^ population. In vivo, erythrocytes were intracellular stained with Ter119, and phagocytosis was measured by Tmem119^+^Ter119^+^ population. Data analyzed by FlowJo; *n* = 4 per group

One of the major functions of any macrophage is phagocytosis and in a hemorrhagic stroke like SAH, red blood cell (RBC) phagocytosis could be beneficial. Using IHC, RBC phagocytosis by microglia in both the 3D human cell culture SAH and in vivo murine SAH models were determined by co-localization of erythrocytes and microglia (Fig. 5A). Both in vitro and in vivo microglial colocalization of erythrocytes suggest microglial phagocytosis of RBCs (Fig. 5A). The yellow co-localization indicates that RBCs are within microglia and being phagocytosed. Furthermore, to the right of each IHC figure is a pixel quantification of the amount of overlap seen between microglia and RBCs. From these data, female from both models phagocytose more RBCs than male SAH, and all SAH models phagocytose more than sham.

While our IHC data support the idea that female microglia phagocytose more RBCs than males, flow cytometry will provide quantitative evidence. Red blood cells from human CSF were treated with a pH-sensitive dye (pHrodo) that increases in fluorescent intensity as the pH decreases; thus, pHrodo-tagged RBCs will emit more fluorescence as they are phagocytosed. In vivo, microglial phagocytosis was quantified by intracellular staining for murine RBCs. Sham controls were exposed to pHrodo alone. No significant pHrodo fluorescence was seen in sham controls, and therefore there was no phagocytosis of the dye alone. Supporting our IHC data, we see increased RBC phagocytosis in female SAH, both in vivo and in vitro, compared to male SAH. The percent of phagocytic microglia in female-SAH brain was almost double that in male SAH in 3D human cell culture SAH and in vivo mouse SAH (Fig. 5B). Thus, both IHC and flow cytometry in the in vivo and in vitro models indicate that female microglia phagocytose more RBCs than males.

### Detection of Efferocytic and Scavenger Receptors on Microglia

**Fig. 6:**
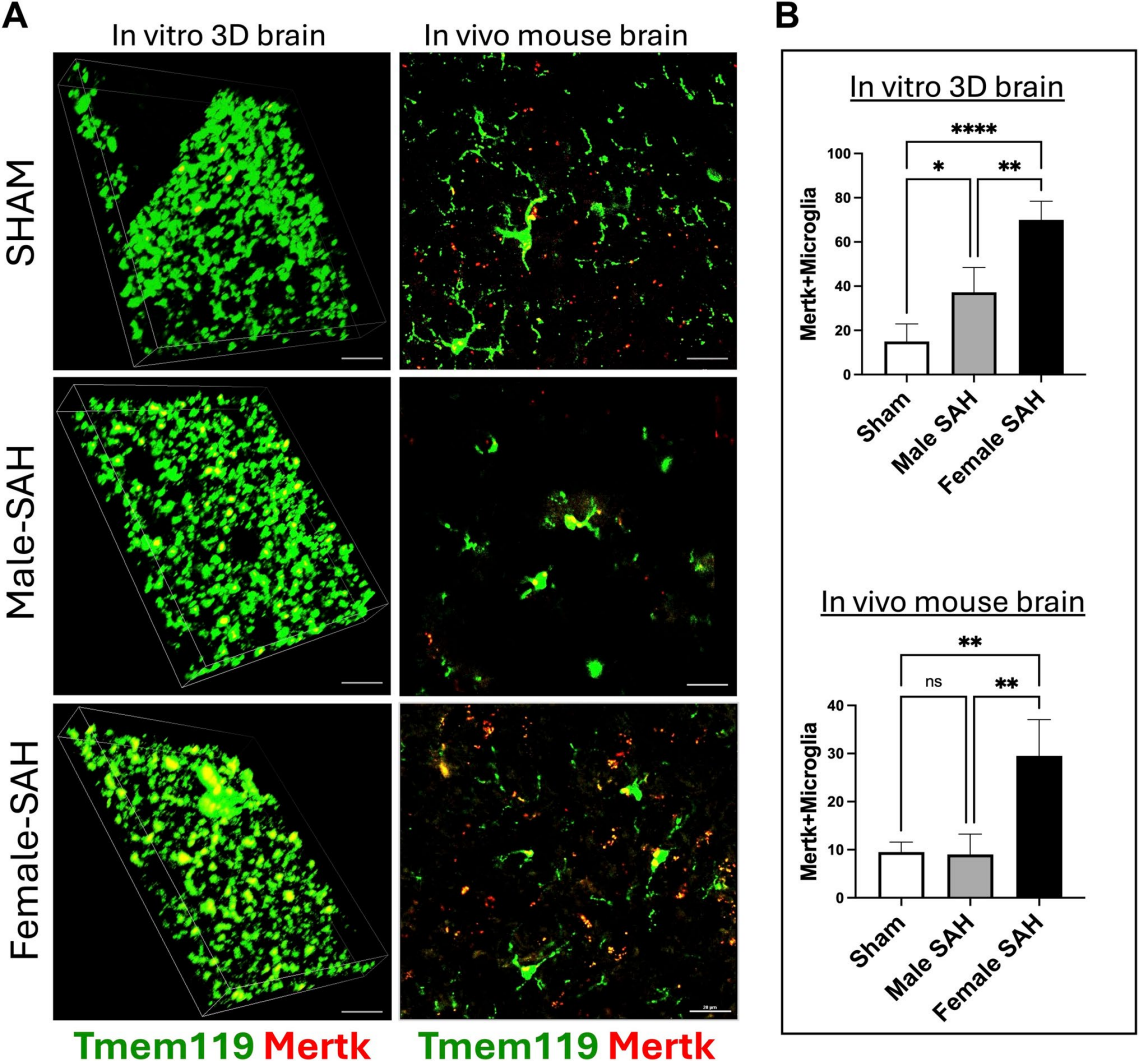

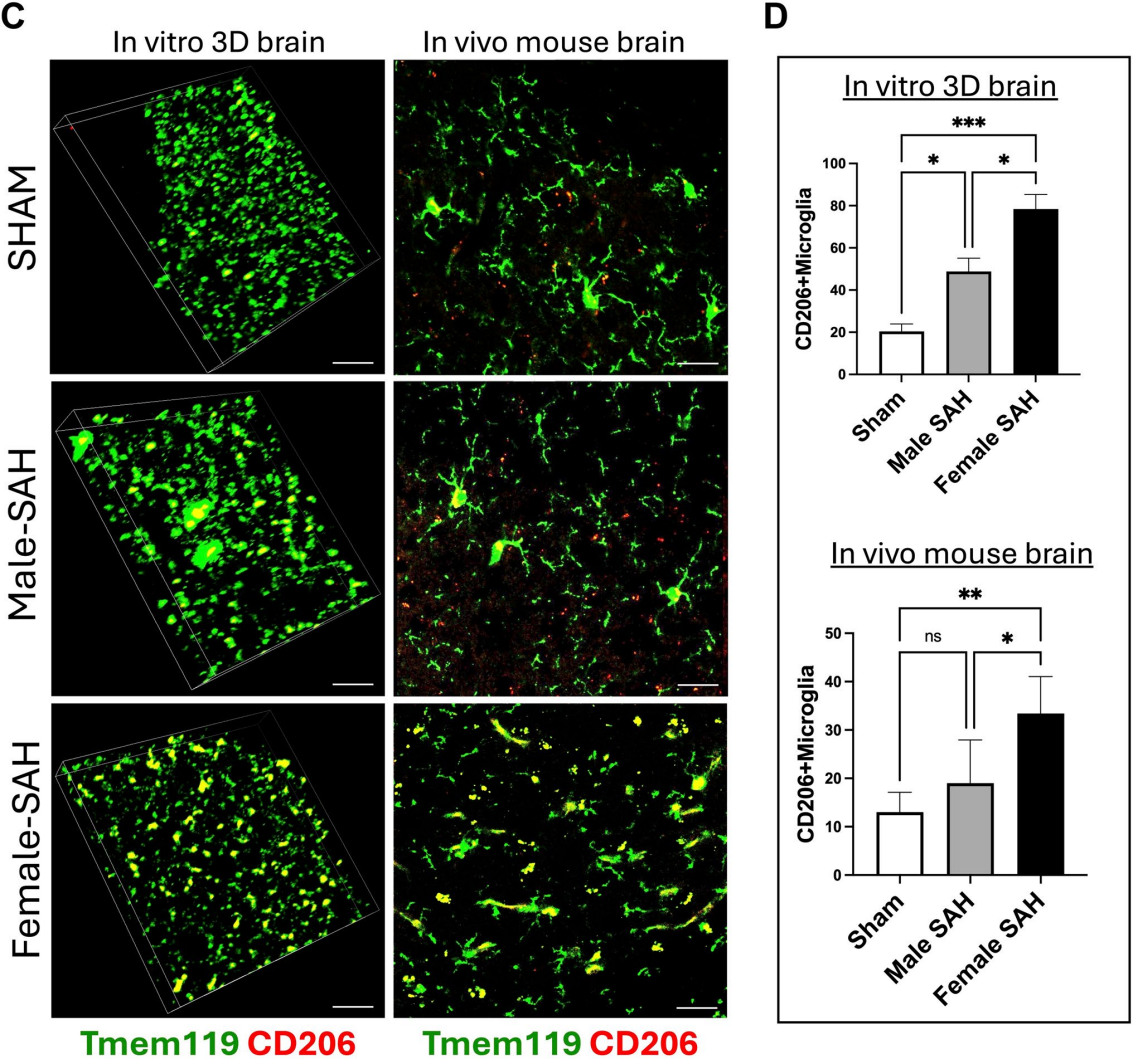
The 3D brain cells and mouse brains were stained with **A** MerTK (red) + TMEM119 (green). **B** Quantification of microglial MerTK expression in vitro and in vivo models by calculating the Tmem119 + MerTK colocalization (yellow) using ImageJ software. **C** Immunostaining of Tmem119 (green) and CD206 (red) in the 3D brain cells and mouse brains. **D** Quantification of microglial CD206 expression in vitro and in vivo models by calculating the Tmem119 + CD206 colocalization (yellow) using ImageJ software. Scale bars = 20 µm; statistical analysis was done by two-way ANOVA, *P < 0.05; *n* = 4 per group, **p <0.01, ***p <0.001, ****p <0.0001, ns: non significant

With our results indicating that a sex-based difference in the phagocytosis of red blood cells existed, we next sought a potential mechanism for this difference. Thus, IHC was performed for MerTK and CD206 on both the 3D human cell culture SAH and in vivo murine SAH models, and co-localization of these receptors with microglia was quantified (Fig. 6A–D). From these data, female from both models express more MerTK and CD206 than male SAH, and all SAH models express more than sham.

### Neuronal Cell Death

**Fig. 7:**
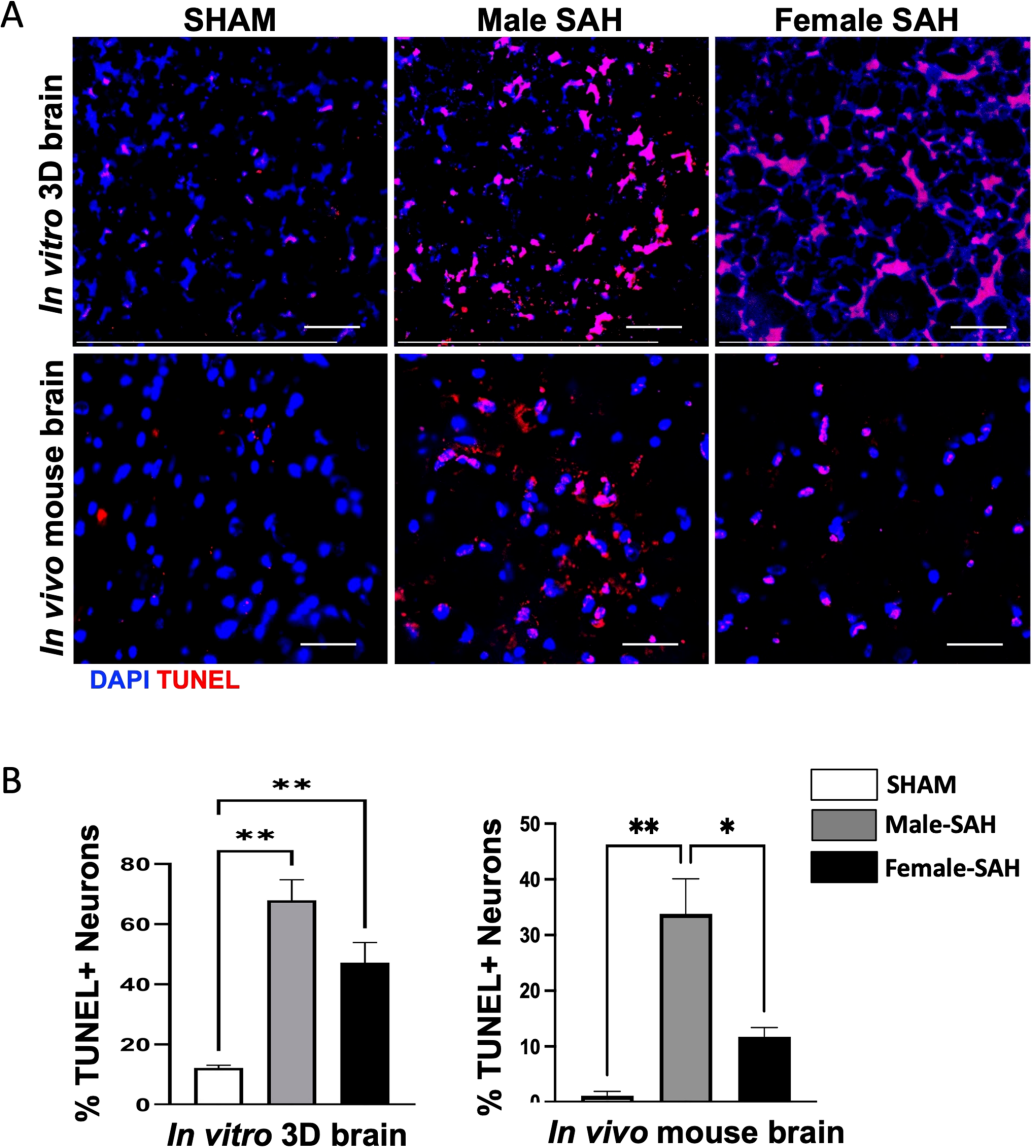
TUNEL assay was performed to detect neuronal apoptosis. **A** Both 3D and mouse brains were counterstained with TUNEL (red) and DAPI nuclei staining (blue). Pink color indicated the TUNEL-positive neurons in the SAH groups. **B** Quantification of TUNEL positive neurons. Apoptotic cell percentage was calculated by measuring intensities of DAPI and TUNEL channels using ImageJ software. Scale bars = 20 µm; statistical analysis was done by one-way ANOVA, *P < 0.05; *n* = 4 per group, **p <0.01

To investigate neuronal apoptosis in our in vitro and in vivo models, we performed TUNEL (terminal deoxynucleotidyl transferase dUTP nick end labeling) counterstained with DAPI. We observed more TUNEL-positive cells in male SAH models than females in both in vitro and in vivo models, indicating greater neuronal apoptosis in males (Fig. 7). Neuronal apoptosis in the male-SAH brain was found to be 1.4-fold higher in 3D cell human culture SAH and threefold higher in vivo mouse SAH than the females (Fig. 7).

## Discussion

Neurological disorders present a substantial burden both in the USA and globally. Our understanding of the pathophysiology of many neurological diseases remains incomplete due to several issues. First, many attempts to study neurological processes are done in a two-dimensional culture system with one or two cell types in co-culture. Cellular interactions change dramatically when comparing 2D to 3D systems as seen in our work (Figs. 1 and 2) and others. Furthermore, by adding the three predominant types of neuroglia in a human brain: astrocytes, neurons, and microglia, the responses seen in our 3D human brain models are more likely to approximate cellular communication and signal transduction in human SAH patients (Fig. 1).

Additionally, the role of the immune system in these models cannot be underestimated. By using individual cerebrospinal fluid samples from SAH patients with external ventricular drains, the leukocytes from whole blood and the macrophages of tissue-resident origin [38] that reside within the CSF can create a very close approximation to the human SAH brain, in vivo, approaching the goal of personalized medicine for the brain.

Our data aligns with findings in murine models of ischemic stroke: indicating that male, murine microglia are more susceptible to inflammation and injury than their female counterparts [39–41]. In our human male microglia, we observed a higher Microglial Morphologic Analysis Index (MMAI), increased interferon-gamma production, and greater neuronal apoptosis (Figs. 2, 4, and 7). This is supported by the fact that no significant difference was seen between the number of microglia in male and female human 3D SAH cultures (Fig. 3). This mirrors the findings in human SAH patients, where female outcomes are slightly better than those of males [14, 17, 22]. Our 3D cell culture brain model suggests that sex-specific innate immune differences contribute to this phenomenon. Similar results were observed in in vivo murine SAH models (Figs. 2, 4, and 7). As a potential explanation for the increased inflammatory nature observed in male SAH, both in the in vitro 3D cell culture and in vivo mouse model, we found that female microglia, in both models, were more effective at erythrophagocytosis (Fig. 5). Furthermore, we investigated a possible mechanism for this phagocytic sex difference and found higher expression of MerTK and CD206 in female microglia (Fig. 6). MerTK is a cell surface receptor tyrosine kinase associated with the clearance of apoptotic cells and red blood cells or efferocytosis [42]. CD206 is a scavenger that works with other receptors like TLR4, CD36, and CD163 to facilitate phagocytosis [43]. This could potentially result in a decreased inflammatory burden and less neuronal apoptosis; however, further experiments are needed to substantiate this hypothesis.

We propose using our “brain-in-a-dish" model as a cost-effective and customizable alternative to animal models. Genetic mutations can be easily introduced and studied in cell cultures, making these models valuable for drug target research. Further research is needed to expand the model’s scope by including other cell types and other neurological diseases.

## Conclusion

In conclusion, our in vitro 3D human brain model showed comparable results to already established in vivo mouse models. Our results demonstrated sex differences in SAH both by 3D, in vitro, human brain models, and murine SAH models. Female 3D brain models and female mouse models of SAH are less inflammatory, less prone to neuronal damage, and more erythrophagocytic than their male counterparts. This offers a novel platform to study sex differences in SAH and possibly other neurologic diseases to develop novel therapeutic options.

## Supplementary Information

Below is the link to the electronic supplementary material.

Supplementary file1 (JPG 1576 KB)

Supplementary file2 (JPG 1930 KB)

Supplementary file3 (JPG 1666 KB)

## Data Availability

No datasets were generated or analyzed during the current study.

## Abbreviations

SAH: Subarachnoid hemorrhage
POD: Post-operative day
CSF: Cerebrospinal fluid
