# Supplementary figures and images for: Development of a 3D Brain Model to Study Sex-Specific Neuroinflammation After Hemorrhagic Stroke
Source: Transl Stroke Res. 2024 Apr 1;16(3):655–71. doi: 10.1007/s12975-024-01243-y (PMC12045812; doi:10.1007/s12975-024-01243-y)

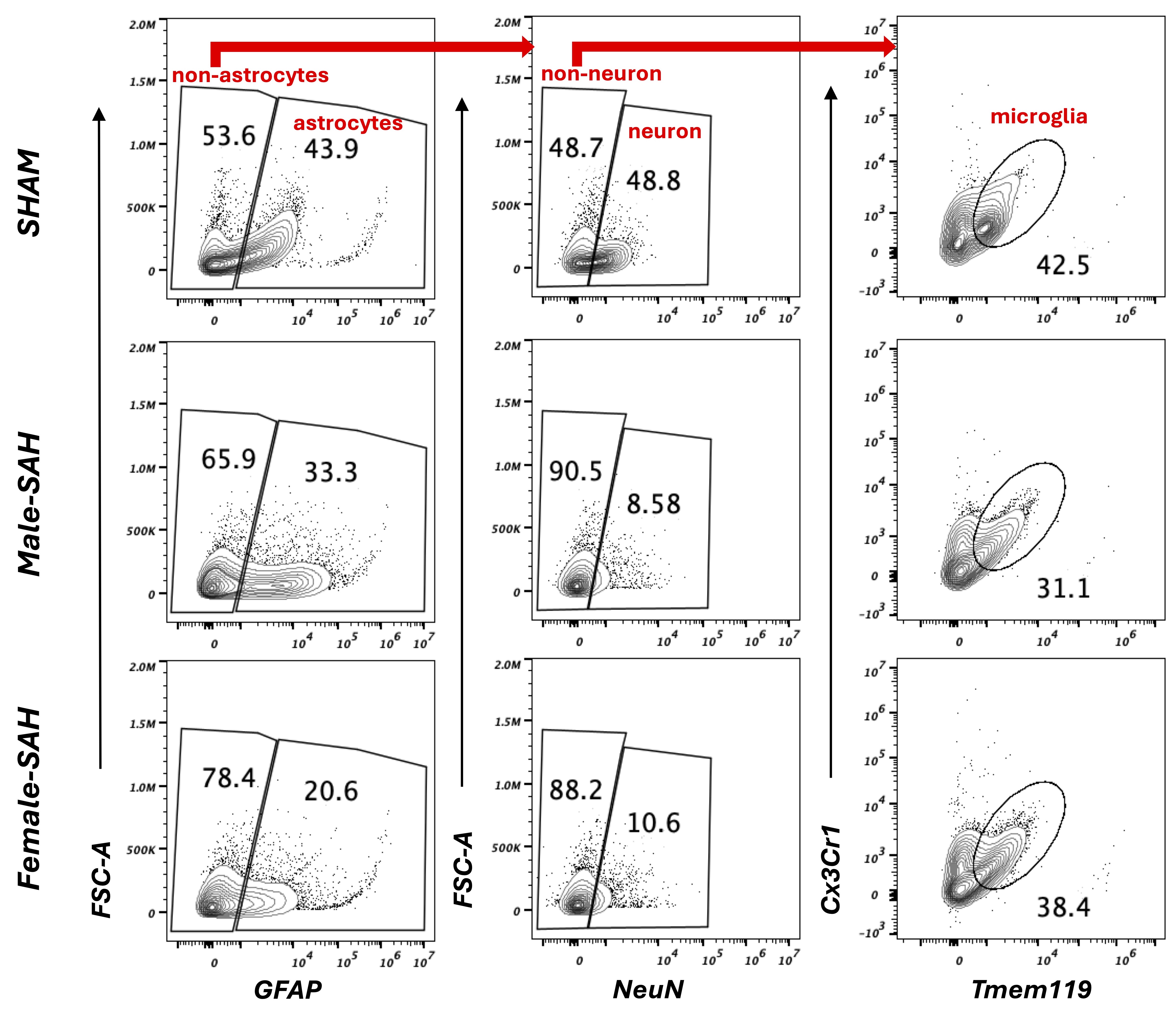

Supplement: Supplementary file 1 — Supplementary file1 (JPG 1576 KB) [file 12975_2024_1243_MOESM1_ESM.jpg]

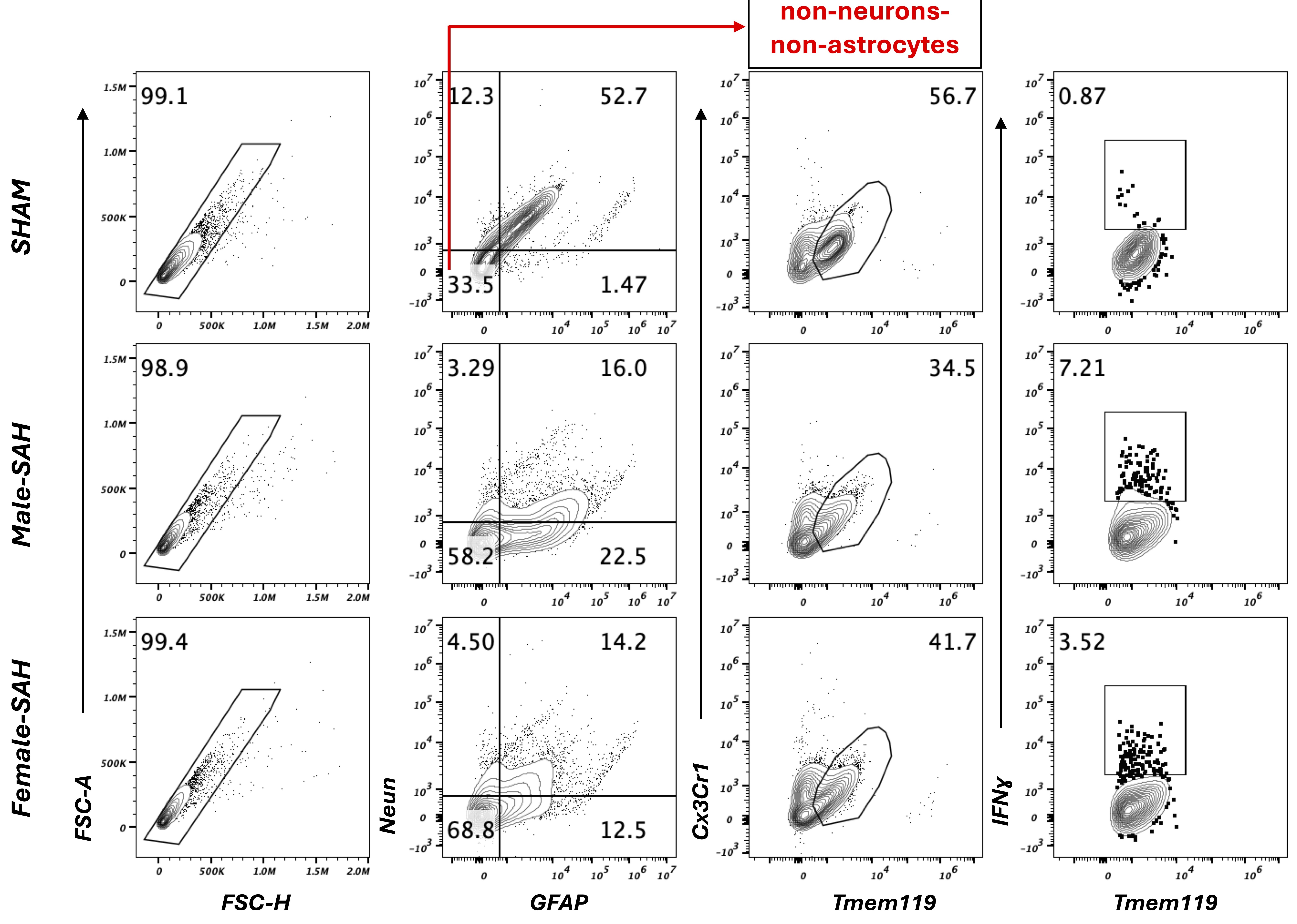

Supplement: Supplementary file 2 — Supplementary file2 (JPG 1930 KB) [file 12975_2024_1243_MOESM2_ESM.jpg]

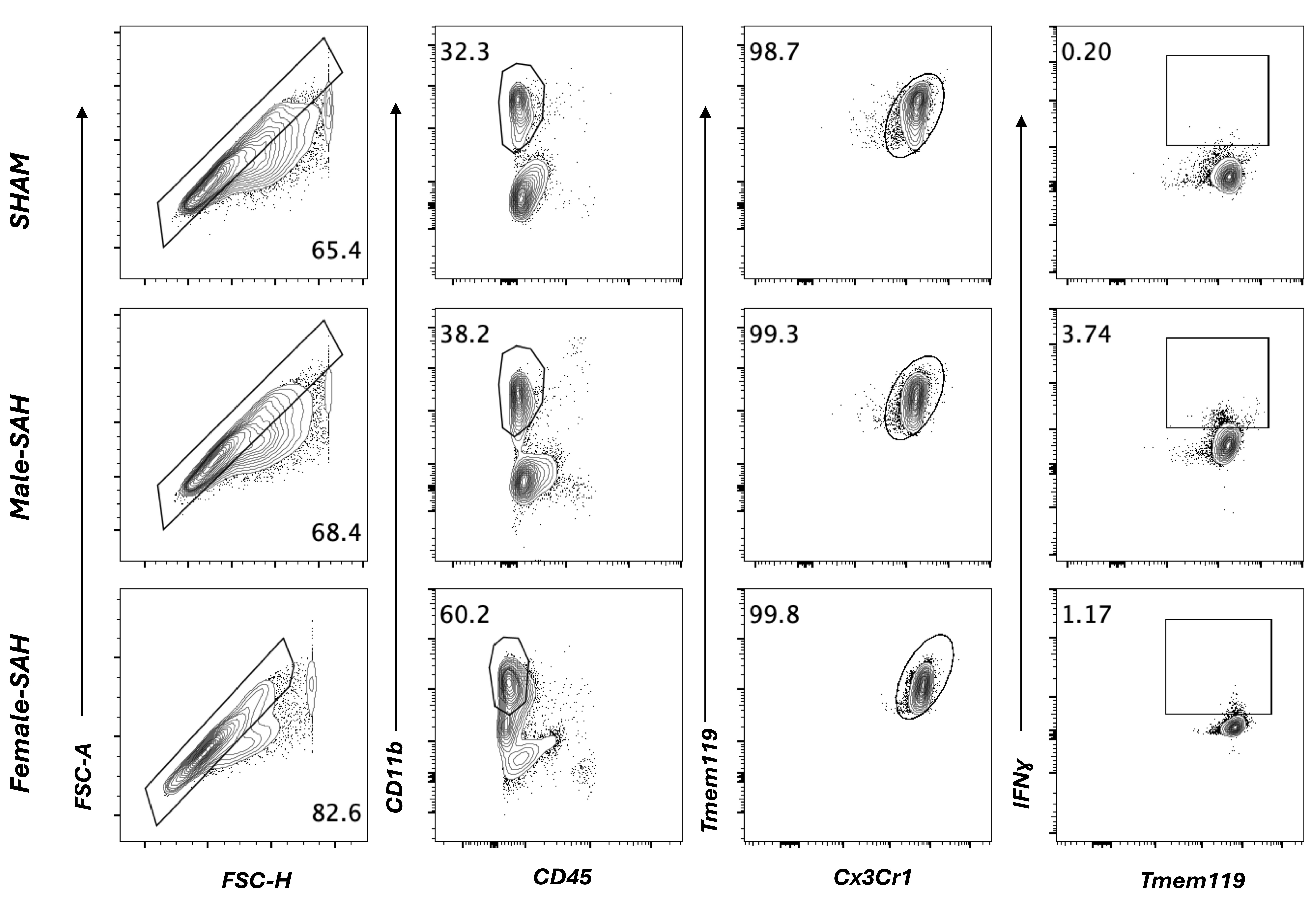

Supplement: Supplementary file 3 — Supplementary file3 (JPG 1666 KB) [file 12975_2024_1243_MOESM3_ESM.jpg]
